# Establishment of Coala: a novel 3D and 2D cancer cell line derived from colorectal cancer liver metastasis

**DOI:** 10.1007/s13577-025-01256-1

**Published:** 2025-08-04

**Authors:** Sandra Mersakova, Juraj Marcinek, Dusan Brany, Olga Chodelkova, Martin Vorcak, Martin Cermak, Martina Poturnajova, Zuzana Kozovska, Henrieta Skovierova, Slavomira Novakova, Barbora Mitruskova, Dusan Loderer, Marian Grendar, Veronika Holubekova, Andrea Hornakova, Jana Melegova, Mariana Brozmanova, Blazej Palkoci, Martin Vojtko, Roman Kycina, Miroslav Pindura, Jan Janik, Peter Mikolajcik, Eva Gabonova, Ludovit Laca, Andreas Nicodemou, Lubos Danisovic, Lukas Plank, Erika Halasova, Marek Mraz, Miroslava Matuskova, Zora Lasabova, Michal Kalman, Jan Strnadel

**Affiliations:** 1 Biomedical Centre Martin, Jessenius Faculty of Medicine in Martin, Comenius University in Bratislava, Mala Hora 4C, 036 01 Martin, Slovakia; 2 Department of Pathological Anatomy, Jessenius Faculty of Medicine in Martin, and University Hospital Martin, Comenius University in Bratislava, 036 01 Martin, Slovakia; 3 Department of Medical Biochemistry, Jessenius Faculty of Medicine in Martin, Comenius University in Bratislava, 036 01 Martin, Slovakia; 4 Clinic of Radiology, Jessenius Faculty of Medicine in Martin, and University Hospital Martin, Comenius University in Bratislava, 036 01 Martin, Slovakia; 5 Department of Genetics, The National Institute of Oncology, Bratislava, Slovakia; 6 Biomedical Research Center of Slovak Academy of Sciences, Cancer Research Institute, Dubravska cesta 9, 845 05 Bratislava, Slovakia; 7 Department of Clinical Genetics, Jessenius Faculty of Medicine in Martin and University Hospital Martin, Comenius University in Bratislava, 036 01 Martin, Slovakia; 8 Department of Pathological Physiology and Central Animal Facility, Jessenius Faculty of Medicine in Martin, Comenius University in Bratislava, 036 01 Martin, Slovakia; 9 Clinic of Surgery and Transplant Center, Jessenius Faculty of Medicine in Martin and University Hospital Martin, Comenius University in Bratislava, 036 01 Martin, Slovakia; 10 GAMMA-ZA, 911 01 Trencin, Slovakia; 11 Institute of Medical Biology, Genetics and Clinical Genetics, Faculty of Medicine, Comenius University, 811 08 Bratislava, Slovakia; 12 Central European Institute of Technology, Faculty of Medicine, Masaryk University, Brno, Czech Republic; 13 Department of Internal Medicine, Hematology and Oncology, University Hospital Brno, Brno, Czech Republic; 14 Department of Molecular Biology and Genomics, Jessenius Faculty of Medicine in Martin, Comenius University in Bratislava, 036 01 Martin, Slovakia

**Keywords:** Colorectal cancer, Liver metastasis, Cancer cell line, 3D culture, In vitro models

## Abstract

Metastatic colorectal cancer is one of the most critical causes of cancer-related dead in patients suffering this type of malignancy. As the liver metastases are found in almost 25–50% of all colorectal cancer patients, there is an urgent need for suitable in vitro and in vivo models. To the best of our knowledge, only a limited number of well-described 2D or 3D organoid/spheroid cell lines originated from human liver metastases was reported for this type of malignancy and submitted to ATCC and other repository centers for general use. Here, we present a novel, well-characterized cell line Coala that was derived directly from liver metastasis of patient with colorectal cancer. Coala cancer cell line was derived by 2D and 3D culture technology and can be, therefore, cultured as standard 2D cell line or as 3D spheroid culture. When transplanted into athymic mice, Coala cell line forms fast growing tumors with histological features similar to original tumor and expressed *CDX2, SATB2,* and *CK20*. Whole exome sequencing analysis of Coala cell line showed the presence of pathogenic mutations in *C8B*, *PRKCQ*, *IRGM*, *FGFR4*, *POLR1C*, *APC* and *TP53* genes. As verified by antibody array chip analysis, Coala cells express *Snail*, *Pdx-1*, *FoxA2,* and *E-cadherin*. Our cell line represents a new, well characterized in vitro research tool for cancer research and will be available to researchers in the field via biorepository centers.

**Supplementary Information:**

The online version contains supplementary material available at 10.1007/s13577-025-01256-1.

## Background

According to WCRFI (World Cancer Research Fund International), colorectal cancer is the 3rd most common cancer worldwide [1]. In 2020, more than 1.9 million new cases were detected. In some countries, the incidence of colorectal cancer has increased over the last decades [2]. Although colorectal cancer is relatively easy to be detected as the symptoms are quite obvious, this type of cancer still kills thousands of patients every year. It is predicted that only in USA; more than 52,900 people will die this year from colorectal cancer [3], mainly from the metastatic spread of primary tumor to surrounding tissues and to distant organs. The most common organ of distant metastasis for colorectal cancer is liver. The character of venous supply to the liver together with immune-tolerance environment of the liver can be one of the explanations of the frequency of colorectal cancer metastasis to this organ [4]. Liver-specific metastasis of colorectal cancer cells can also be explained by their expression of some chemokines and by responding of colorectal cancer cell-expressed receptors to specific homing signals from liver (CXCL12). Metastatic organotropism and the escape of cancer cells from primary tumor site to the metastatic site are also connected with process of epithelial-mesenchymal transition (EMT) [5].

A quite limited number of well characterized metastatic colorectal cancer-derived cell lines are currently available for researchers (Table 1). Moreover, the cancer cell lines that are capable of growing as organoid/spheroid culture are preferred as according to studies they capture the heterogeneity and genetic features of the original tumors. These cell lines are also considered to be better models for predicting treatment responses [6–8]. Therefore, there is an urgent need for novel in vitro and in vivo models for this cancer [9, 10]. Here, we report the establishment and characterization of a novel cancer cell line derived from liver metastasis of patient with colorectal cancer. The cell line was derived with optimized protocol published previously by our group [11]. Coala cells showed aggressive phenotype in vivo when xenografted into athymic nude mice; in vitro Coala cells form tumorspheres and show the membrane expression of several typical CD markers. When compared, Coala cells share three pathological mutations with the original liver metastasis.

**Table 1:** The summary of organoid culture and 2D colorectal cancer cell lines in repository centers

ATCC—American Type Culture Collection
Colon cancer cell models derived from metastatic colon cancer
All cell models are organoids derived from primary patient samples
These cell models are patient-derived next-generation cancer model generated by the Human Cancer Models Initiative (HCMI)

No.	Cell model name	ATCC no.	Cell model
1.	HCM-SANG-0265-C18	PDM-42	Organoids derived from primary patient samples
2.	HCM-CSHL-0736-C18	PDM-425	Organoids derived from primary patient samples
3.	HCM-CSHL-0726-C18	PDM-419	Organoids derived from primary patient samples
4.	HCM-CSHL-0653-C18	PDM-410	Organoids derived from primary patient samples
5.	HCM-CSHL-0603-C18	PDM-372	Organoids derived from primary patient samples

Leibniz Institute DSMZ—Deutsche Sammlung von Mikroorganismen und Zellkulturen
Colon cancer cell models derived from liver metastasis
All cell models are patient-derived 2D cell lines

No.	Cell model name:	DSMZ no.	Cell model
1.	JVE103	ACC 807	2D cell line
2.	JVE114	ACC 812	2D cell line
3.	JVE187	ACC 818	2D cell line
4.	JVE253	ACC 823	2D cell line
5.	JVE371	ACC 825	2D cell line

RIKEN—Bioresource Research Center, Japan	
JCRB—Japanese Collection of Research Bioresources Cell Bank	
No colon cancer cell models derived from liver metastasis	

## Materials and methods

### Clinical case

A 68-year-old man underwent (in 2020) an extensive right-sided hepatectomy due to presence of metastases (after previous sigmoid colectomy, performed in 2013) for clinical stage I colorectal adenocarcinoma (grade G3, L1, V1, B0, Pn1). Chemotherapy treatment was based on intravenous infusion of combination of irinotecan, oxaliplatin, calcium folinat and 5-fluorouracil (FOLFOXIRI). Patient signed informed consent letter, previously approved by the Ethical Review Committee of Jessenius Faculty of Medicine in Martin and donated residual tumor tissue for research. The postoperative course was without complications, the patient was discharged from hospital (University Hospital Martin, Slovakia) in good condition.

### Establishment of new cancer cell line

Piece of viable tumor tissue (residual part from histological examination) was transferred from Department of Pathological Anatomy, in ice-cold culture medium (DMEM/F12 containing 10% FBS and 1% P/S) into the lab. Tumor was then processed in accordance with the protocol, published previously by our group [11]. Briefly, single-cell suspension was prepared from tumor tissue with collagenase IV and washed twice with PBS. The cells were then plated into Petri dishes and observed daily for epithelial cell-colony formation. When colonies were detected and expanded, they were embedded into Matrigel matrix and plated into ultra-low attachment 6-well plates (Corning, USA) as described [11]. Coala cell line was also shown to be able to grow in 3D culture conditions in form of Matrigel matrix-free floating spheroids. When placed back into standard, tissue culture-treated polystyrene plates, Coala cells continued to grow in form of 2-dimensional, sharp-edged colonies.

### Histological analysis (histology)

For histological analysis, tumor extracted from patient, or tumor tissue from human cancer cell-xenograft were fixed in 10% paraformaldehyde (Cell Signaling, USA) for 72 h and then processed with standard procedures (graded ethanol dehydration followed with paraffin embedding of tissue). Five-micron-thick sections from paraffin blocks were used for the bright-field microscopy examination of tissue specimens. The list of antibodies, used for staining, is indicated in Supplementary Fig. 2*.*

### STR analysis (DNA fingerprinting)

Coala cancer cell line was tested for the originality and possible contamination with other cells with ATCC Human Cell STR profiling service. The original tumor cells, isolated from the fresh tumor tissue were also included for the analysis to prove the same origin of tumor tissue sample and Coala cell line.

### Mycoplasma testing

Commercial PCR Mycoplasma test kit (ATCC, USA) was used for detection of possible mycoplasma infection of Coala cells. Briefly, the cells were grown in the dish and processed at desired confluency as recommended by manufacturer. Standard PCR (with the detection of DNA on 2% agarose gel) was run for the detection of more than 60 species of mycoplasma, acholeplasma, spiroplasma, and ureaplasma. As a negative control, sample from commercially available cell line (mouse embryonic fibroblasts, and millipore) was included in the test. Negative PCR control was also included. Positive control from the ATCC kit and positive control mixed with sample from Coala cells were also added to the run.

### Karyotype analysis and FISH

The chromosomes derived from cultured Coala cells were analyzed by standard cytogenetic G-banding method. This analysis revealed various numerical and structural aberrations including deletions and translocations. A specification of involved chromosomes was beyond the limits of standard cytogenetic G-banding procedure (Supplemental Fig. 1). For this reason, the multicolor FISH method (M-FISH with 24Xcyte Human Multicolor FISH probe (MetaSystems, Germany) was used. Fluorescent microscope Olympus BX51 (Olympus Optical, Japan) and ISIS imaging system (MetaSystems, Germany) were used for imaging.

**Fig. 1:**
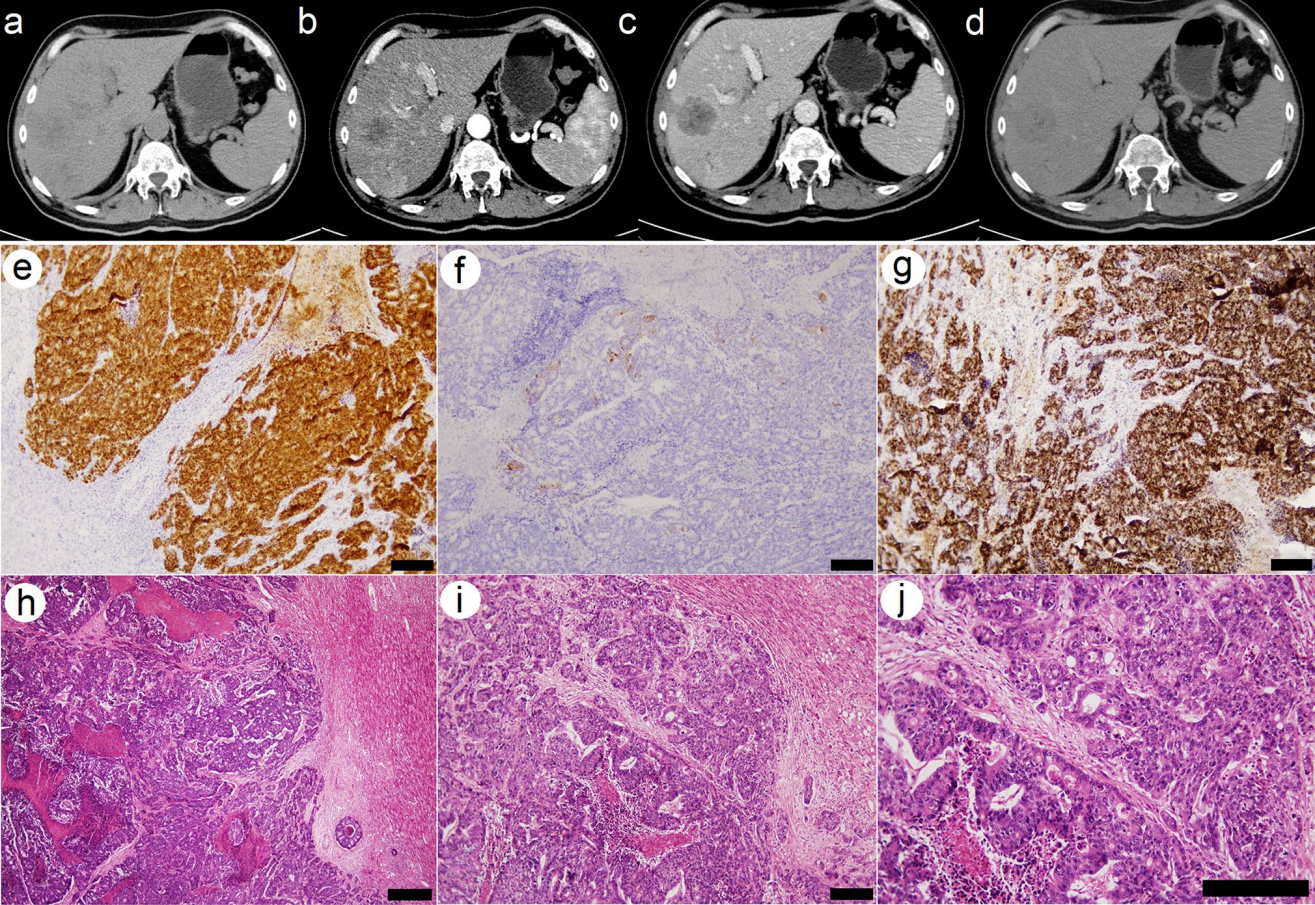
CT (computed tomography) scans of the liver in a patient with MTS involvement in primary CA of the sigmoid colon. CT shows multiple foci of the right lobe of the liver (depicted lesion in S8)—mildly hypodense non-enhanced scan (**a**), contrast enhanced scan at the arterial phase with peripheral hyper density (**b**), hypodense at the portal (**c**) and delayed phase (**d**). Image **e** shows positive staining for CDX2 homeobox protein in liver metastasis and **f** shows Cytokeratin 20 staining. Positive staining for SATB2 (Special AT-rich sequence-binding protein 2) is shown in image **g**. Hematoxylin/eosin staining (**h**–**j**) of metastatic tumor tissue in liver is also shown in this figure. Scale bar represents 50 µm

### Flow cytometry analysis of surface markers

**Fig. 2:**
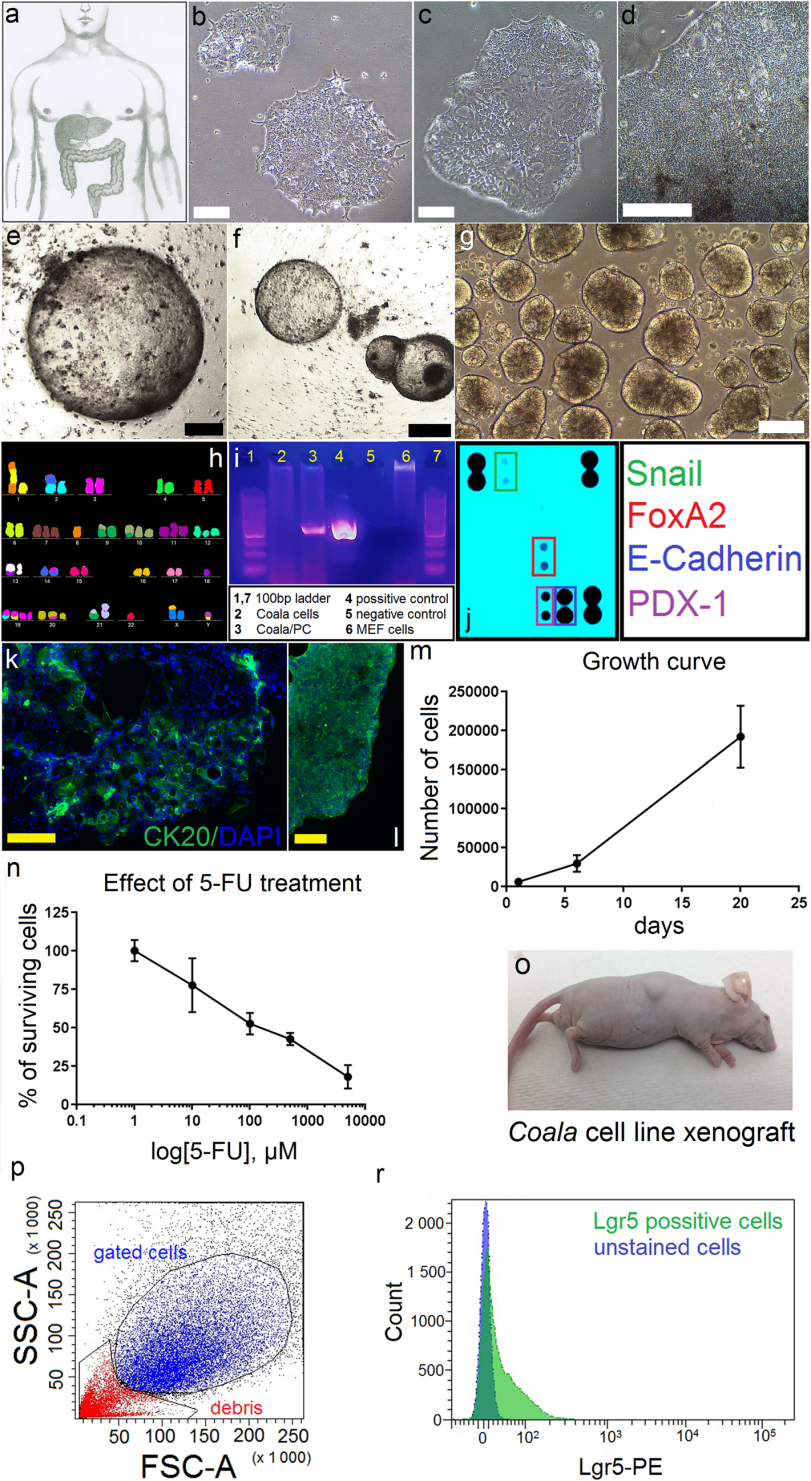
Characterization of newly-derived Coala cancer cell line. Cell line was derived from human colorectal cancer liver metastasis (**a**), forms sharply-edged, fast growing colonies in 2D (**b**–**d**), when embedded into Matrigel matrix, the cell line form spherically-shaped tumorspheres (**e**, **f**). Image **g** shows the phenotype of tumorspheres, when Coala cells are cultured in ultra-low attachment plates. Scale bars represent 50 µm (**b**–**d** and **k**, **l**) and 500 µm (**e**–**g**). Multicolor (M-FISH) analysis of Coala cells showing the abnormal karyotype (**h**). Negative PCR test (detection of multiple mycoplasma species) in Coala cell culture (**i**). Antibody array chip analysis shows the expression of Snail, FoxA2, E-Cadherin and Pdx-1 in Coala cell lysate. Cultured Coala cells show positivity for Cytokeratin 20 (DAPI was used for nuclei staining), scale bar represents 50 µm. Growth curve for Coala cells (**m**) and effect of 5-Fluorouracil treatment (**n**). Image (**o**) showing a tumor formed in athymic mouse 3 weeks after subcutaneous grafting of Coala cells. Images (**p**) and (**r**) show flow cytometry analysis of Lgr5 marker in early passage (P3) of Coala cells. Cells from passage No.21 were used in experiments

For surface marker screening, the standard flow cytometry with FACS Aria II cytometer (BD Biosciences, USA) for verification of Lgr5 and selected CD markers was used. We also used multiplex flow cytometry (designed for screening majority of CD markers). MACS Marker Screen (MiltenyiBiotech, Germany) plate kit and MACSQuant Instrument allowed screening of 376 human surface CD markers on newly derived Coala cancer cell line. The cells were prepared in accordance with manufacturer´s instructions and analyzed with MACSQuant instrument (MiltenyiBiotech, Germany), using the 640 nm and 488 nm lasers. Gating was used to exclude dead cells and debris from the analyses (Fig. 2p).

### Xenotransplantation of human cancer cells

**Fig. 3:**
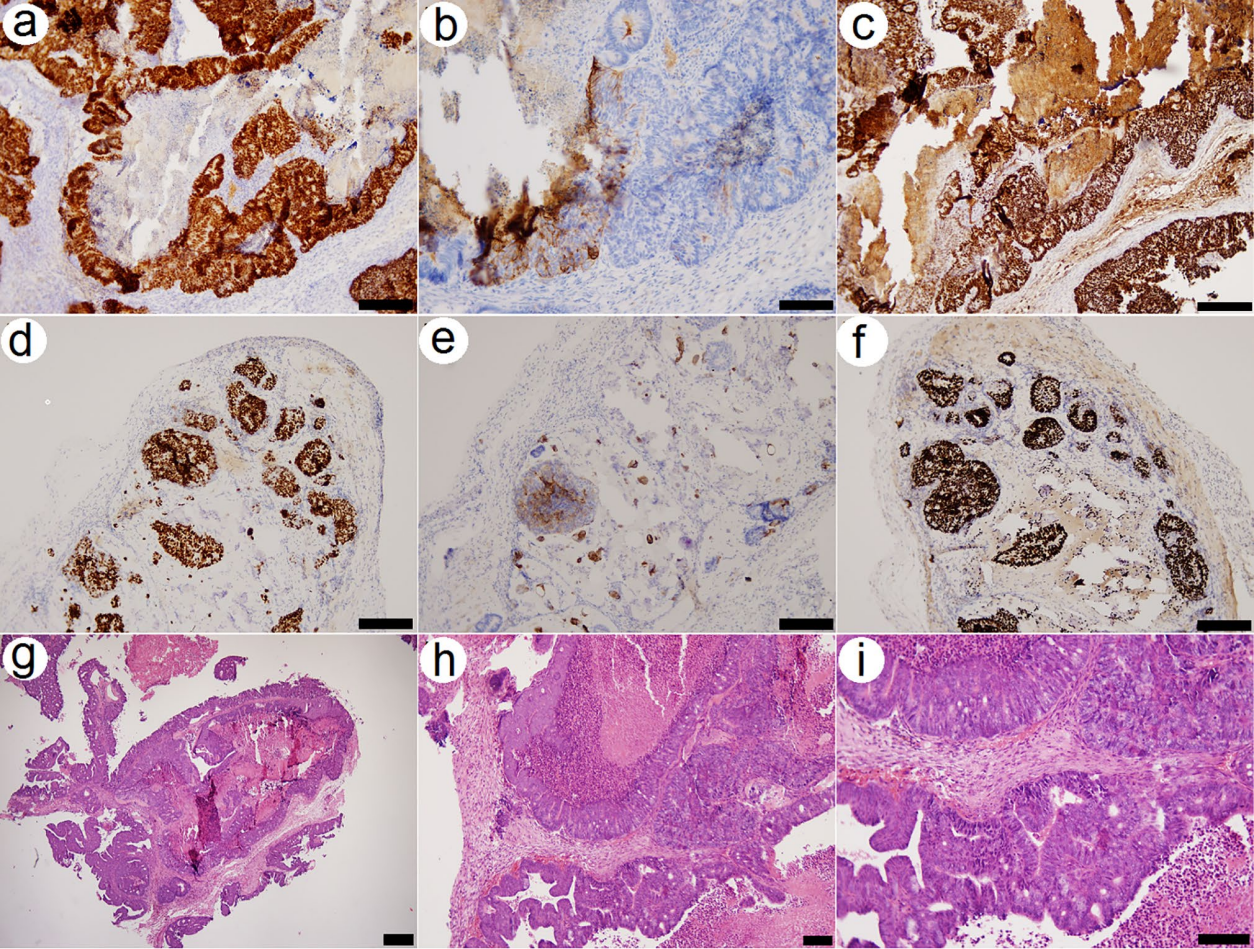
Immunohistology of Coala cancer cell line xenograft shows positive staining for CDX2 (**a**, **d**), Cytokeratin 20 (**b**, **e**) and SATB2 (**c**, **f**). Images (**f**–**h**) show hematoxylin/eosin staining of human Coala cancer cell line-derived xenograft tissue, isolated from athymic mice. Cells from passage No.21 were used in the analysis. Scale bar represents 50 µm

The in vivo ability of Coala cancer cells to form tumors, histologically similar to original human tumors was tested with xenotransplantation of cells into 7-week-old Crl:NU(NCr)-Foxn1nu athymic (nude) mice (The Jackson Laboratory, USA). Briefly, the mixture of cells (2 × 10^6^ cells/injection) and 30% Matrigel (Corning, USA) diluted in ice-cold PBS (200 µL, pH 7.4) was injected subcutaneously (into the right flank) with insulin syringe (precooled in ice-cold PBS). Animals (*n* = 3) were then periodically checked for tumor formation. Palpable tumors (Fig. 2o) were detected approx. 3 weeks after injection of cells. Before tumor extraction, the animals were euthanized by cervical dislocation. Tumor tissue was fixed in 10% paraformaldehyde and processed as described previously [12]. Immunohistology proved the similarity of tumor xenograft with original tumor (Fig. 3a–i). The animal experiments were approved by Ethical Review Committee of Jessenius Faculty of Medicine in Martin, Comenius University in Bratislava, and The State Veterinary and Food Administration of the Slovak Republic (Approval No. 4987-3/2022-220).

### Next generation sequencing

For primary human tumor mutation analysis, the TruSight™ Oncology 500 (Illumina, USA) targeted, hybrid-capture based next-generation sequencing assay was used to analyze 523 genes (assessing all DNA and RNA variants types) for mutations. Sample preparation was in accordance with manufacturer’s instructions. Data analyses were performed in RUO mode of NextSeq 550DX instrument (from the same company), using the TSO500v2.0.0 Local App. Analysis (customized) pipeline from Clin. Genomics Workspace software from Pierian Dx was adapted in this project.

### Whole exome sequencing

To identify the genetic variants (altering the protein sequences), whole exome sequencing (WES) experiment was performed on DNA sample, extracted from cultured Coala cells for sequencing the protein-coding regions. Kapa HyperPlus Kit, HyperCap Target Enrichment Kit, HyperCap bead Kit and HyperExome Probes were used for sample preparation and exonic DNA sequencing. The samples were then analyzed with Illumina HiSeq (Illumina, USA) instrument.

### Immunocytochemistry

Immunocytochemical analyses of selected proteins were performed with Coala cells, grown on plastic microscopy chambers (Ibidi, USA). Cells were fixed in 4% paraformaldehyde (Cell Signaling, USA) and processed as described previously [12]. Olympus IX71 fluorescent microscope (Olympus, Japan) and ImageJ software was used for image processing and figure preparation.

### Antibody array

Expression of 19 transcription factors was detected with Stem Cell Antibody Array (RD Systems, UK). The Coala cells were processed according to manufacturer´s recommendation and chemiluminescence detected with Blue X-ray photosensitive film (Kodak, USA). Scanned images were then analyzed with ImageJ software.

### Doubling time

Briefly, the cell number was calculated with Biorad T20 cell counter and cells were seeded on Petri dishes and cultured for 20 days. Doubling time of Coala cancer cells was estimated to be around 2,5 day as calculated with doubling time calculation formula (doubling time = [*t* × (ln2)]/ [ln(*N*20/*N*0)].

### Sensitivity of Coala cancer cell line to 5-fluorouracil

The sensitivity of Coala cancer cell line to 5-fluorouracil was evaluated by cell viability assay. Cells were plated on the six well plates and exposed to different concentrations of 5-fluorouracil (range from 0.1 to 10,000 µM of 5-fluorouracil). Percentage of surviving cells was counted and plotted on *y*-axis. Data are presented as means ± standard error of the mean (SEM) of quadruplicate analyses.

### Pyrosequencing analysis

Coala cell line sample treated with sodium bisulfite also underwent pyrosequencing analysis for four selected genes. During the PCR reaction, cytosine residues were converted to uracil or thymine in the final PCR product, while methylated cytosines remained unchanged. We focused on identification of methylated regions within the sequences of four genes: *CDKN2A*, *PROM1*, *MGMT*, and *ADAMTS16*. As a control, completely methylated and completely unmethylated DNA was used. We analyzed 7CpGs for *MGMT*, 6CpGs for *CDKN2A*, 4CpGs for *PROM1* and 3CpGs for *ADAMTS16*. For the analysis of the selected regions of *PROM1* (4 CpG), *CDKN2A* (6 CpG), *ADAMTS16* (3 CpG) and *MGMT* (7 CpG), commercially available CpG assays [PyroMark CpG Assay (200), URL address: https://geneglobe.qiagen.com/product-groups/pyromark-cpg-assays: Hs_*PROM1*_05_ PM (Cat. No. PM00110194), Hs_*ADAMTS16*_01_PM (Cat. No. PM00022106), Hs_ *MGMT*_01_PM (Cat. No. PM00149702) and Hs_*CDKN2A*_02_PM (Cat. No. PM00039907)] were used.

### Evaluation of metastatic potential

The in vivo study was approved by the Institutional Ethic Committee and by the State Veterinary and Food Administration of Slovak Agency under approval No: Ro-290-3/2020-220. Seven-week-old NSG® mice (NOD.Cg-PrkdcSCID IL2rgtm1Wjl/SzJ, Jackson Laboratories USA) were used under institutional guidelines and approved protocols. The approx. 7.5 × 10^6^ cells in 150 μL of 1:1 ECM/DMEM mixture were bilaterally administered subcutaneously into the flanks. Four mice were used for this experiment. Xenografts were measured by caliper three times a week, and volume was calculated using the following formula: *V* = 0.5236 × ((width + length)/2)^3^. Animals were euthanized after 34 days. Xenografts were excised and weighed, and mice were checked for visible metastasis. Xenografts, lungs, liver, and spleen were subjected to molecular analysis to detect micrometastases.

### qPCR analysis

Genomic DNA was isolated by innuPREP DNA Kit (IST Innuscreen, Germany) using the manufacturer’s instructions. Human DNA sequences in mouse tissues were searched by quantitative PCR (qPCR) using GoTaq® Probe qPCR (Promega, USA) and thermocycler CFX 96 (Bio-Rad, USA). 200 ng of purified DNA was amplified in qPCR using primers and probes specific for mouse *RAPSYN* (receptor-associated protein of the synapse) gene and human *β-GLOBIN* gene (Supplementary Table 4).

The DNA isolated from mouse cell line NIH/3T3 (ATCC® CRL-1658™) was used as a positive mouse control. The DNA isolated from human cell line HT-29/FURiv-met (https://pubmed.ncbi.nlm.nih.gov/30143021/ and DNA from lung metastasis of human colorectal cancer (PK3) were used as positive controls for presence of human DNA.

The qPCR program was set up as follows: 95 °C initial denaturation for 2 min followed by 40 cycles at 95 °C for 20 s, 60 °C 1 min and then the plate was read. Final annealing was 10 min at 60 °C followed by a cooling step (7 °C). All samples were run in three or four technical replicates, in three independent experiments.

## Results

### Tumor histology

Macroscopically, the tumor isolated from liver metastasis (Fig. 1a–d) was significantly necrotic (about 50% of the volume), with the vital structures represented by a cribriform and tubular growth pattern (Fig. 1h–j). The tumor cells were cylindrical and polygonal with eosinophilic cytoplasm also with rare intraluminal deposits of mucin. The nuclei were vesicular with distinct nucleoli. Immunohistochemically, the tumor showed expression of *CDX2*, *CK20* and *SAT-B2* (Fig. 1e–g). Expression of *Cytokeratin 20* was also verified with immunocytochemistry (Fig. 2k, l); the list of antibodies used is shown in Supplementary Fig. 2). The tumor propagated into blood and lymphatic vessels, without obvious intra ductal spread and without perforation of the superficial serosa. The original liver parenchyma was free of pathological changes.

### Coala cancer cell line derivation

Isolated primary cancer cells grew in the form of small colony-like 2D structures (Fig. 2b–d) when standard culture plates were used, and also made typical, spheroid 3D structures, when grown in ultra-low attachment plates (Fig. 2g) or when embedded in Matrigel (Corning, USA) matrix gel (Fig. 2e, f). In 2D cultures, remaining tumor stromal cells were detected for several passages. The colorectal cancer cells were, therefore, isolated by manual picking of cancer colonies with the pipette (before picking, the position of cancer cell colonies was marked under microscope with permanent marker) and transferred into new culture plates. Cells were continuously cultured for more than 30 passages and cell aliquots from every passage were stored in freezing medium (10% DMSO and 90% culture medium). Mr. Frosty container filled with isopropanol was used for gentle cell freezing. Frozen aliquots were then transferred from − 80 °C freezer into Dewar container and stored in vapor phase of liquid nitrogen.

### Recovery of Coala cells after freezing in liquid nitrogen

Interestingly, after plating the cells from freshly de-frosted frozen aliquots, the recovery of cells took approximately 2–3 days until the appearance of small, sharp-edged colonies was observed (Fig. 2b–d)*.* To increase the recovery of cells after defrosting cryovials from liquid nitrogen, the addition of 10 μM ROCK inhibitor (Y-27632, StemCell Technologies) to the cell culture medium is recommended. During routine passaging, the addition of ROCK inhibitor is not essential.

### FISH analysis

The multicolor FISH method (M-FISH) was used to characterize the Coala cell line and composite karyotype, which contains all clonally occurring abnormalities, was compiled as follows:51~54,X,der(X)t(X;16),der(Y)t(Y;1).ish,der(1)t(8;1;8),der(1)del(1p?),der(2)t(X;2),der(2)del(2q?),+7,t(9;10),+10,+11,+der(12),der(13)t(13;1),−18,+19,+19,der(19)t(1;19)[cp6] (Fig. 2h).

### Mycoplasma test

PCR-based mycoplasma test for detection of more than 60 species of mycoplasma, acholeplasma, spiroplasma and ureaplasma proved that Coala cancer cell line was free of mycoplasma infection (Fig. 2i)*.*

### Analysis of transcription factors by antibody array

Human pluripotent stem cell antibody array analysis of 15 stem cell markers expression indicated that Coala cancer cell line express high levels of *E-Cadherin*, *Fox-A2* and *PDX-1* and moderate levels of *Snail* transcription factor (Fig. 2j)*.*

### Growth curve analysis

Doubling time of Coala cells was estimated to be around 2.5 day as calculated with doubling time calculation formula (doubling time = [*t* × (ln2)]/[ln(*N*20/*N*0)] (Fig. 2m).

### Sensitivity to 5-fluorouracil

Coala cancer cell line was shown to be sensitive to 5-fluorouracil as shown in Fig. 2n.

### Xenotransplantation of human cancer cells

Xenografting of human colorectal cancer cells into athymic mice proved the ability of newly derived human cancer cell line to induce fast-growing tumors, histologically similar to original tumor, isolated from patient (Fig. 3a-i)*.*

### Flow cytometry analysis of surface markers

Multiplex flow cytometry analysis of 376 human surface CD markers on newly derived Coala cancer cell line using MACS Marker Screen (Miltenyi Biotec, Germany) plate kit and MACSQuant Instrument showed that high percentage (more than 90%) of Coala cells express CD9, CD26, CD29, CD49B, CD49f, CD66acde, CD66ace, CD98, CD147, CD164 and CD326. Some other markers were also abundant—for full list of markers detected and % of positive cells see the Supplementary Fig. 3, Supplementary Fig. 4 and Supplementary Table 3). Interestingly, *Claudin-3* was found to be expressed in 58.15% of cells, and *SSEA-4* in 61.33% of cells. No expression was detected for few other markers (e.g., CD21, CD22, CD61, and CD69). Interestingly, the expression of *Lgr-5* was found to be expressed in freshly-isolated cancer cells (Fig. 2p–r), but not in Coala cancer cell line at later passage (P21) (Supplementary Fig. 3, Supplementary Fig. 4 and Supplementary Table 3).

### STR fingerprinting analysis

Based on STR fingerprinting analysis*,* Coala cell line was proved to be human, original and free of other cell type contamination as verified by comparing with the ATCC (American Type Culture Collection) reference database profile. STR fingerprinting also verified the same origin of tumor sample and isolated Coala cell line. *Note:* To protect the identity of the donor, only selected *loci* are shown as recommended by ATCC (Supplementary Fig. 5)*.*

### Whole exome sequencing

Whole exome sequencing of Coala cancer cell line discovered at least 7 mutations with clinical significance described as “pathogenic” (Supplementary Table 1, orange color) and several mutations that can be described as “likely pathogenic” (Supplementary Table 1, yellow color)*.* The pathogenic mutations were found in *C8B* (Complement component 8), *PRKCQ* (gene encoding Protein kinase C theta), *IRGM* (Immunity-related GTPase family M protein), *FGFR4* (Fibroblast growth factor receptor 4), *POLR1C* (RNA polymerase I and III Subunit C), *APC* (Adenomatous polyposis coli) and *TP53* (tumor protein 53) genes (Table 2 and Supplementary Table 1, orange color)*.*

**Table 2:** The summary data and the sequencing statistics for TruSight™ Oncology 500 targeted, hybrid-capture based next-generation sequencing analysis

A. Detailed information of mutations from report (variants from tiers IA, IB, IIC and IID)							
Gene	DNA change	AA mutation	CDS mutation	Classification	Transcript—NCBI ID	VAF	Depth
*TP53*	chr17:g.7577538C>T	p.R248Q	c.743G>A	IB	NM_000546.5	50.4	1250
*APC*	chr5:g.112175218_112175222del5	p.E1309Dfs*4	c.3927_3931del5	IIC	NM_000038.5	20.7	1333
*APC*	chr5:g.112175354delT	p.S1355Lfs*60	c.4063delT	IIC	NM_000038.5	36.8	1636
*BRCA1*	chr17:g.41244000T>C	p.K1183R	c.3548A>G	IIC	NM_007294.3	22.9	1629
*ERBB2*	chr17:g.37884037C>G	p.P1170A	c.3508C>G		NM_004448.2	20.6	1454
*FGFR4*	chr5:g.176520243G>A	p.G388R	c.1162G>A	IIC	NM_213647.1	61.1	529
*NOTCH1*	chr5:g.176520243G>A	p.R912W	c.2734C>T	IIC	NM_017617.3	86.2	817
*TP53*	chr17:g.7579472G>C	p.P72R	c.215C>G	IIC	NM_000546.5	99.9	1642
*ASXL1*	chr20:g.31022959T>C	p.L815P	c.2444T>C	IID	NM_015338.5	100.0	3512
*PARP1*	chr1:g.226555302A>G	p.V762A	c.2285T>C	IID	NM_001618.3	38.1	1226
*RET*	chr10:g.43613908A>T	p.Y791F	c.2372A>T	IID	NM_020975.4	38.6	1356

B. The sequencing statistics of TSO500 analysis	
Total number of reads	66,635,811
% Mapped reads	99.56%
On target reads	55,372,700
% On target reads	83.47%
Number of UMI families	43,770,836
% Unique on target reads	79.05%
% Positions > 50 × unique	98.67%
% Positions > 250 × unique	95.60%
Average unique coverage	1284.0

C. Detailed information of CNVs from report (CNVs from tiers IA, IB, IIC and IID)			
Gene	CNV type	Copy number	Classification
CCND3	Copy number gain	3 copies	IIC
AKT2	Copy number gain	5 copies	IID
BRCA2	Copy number gain	4 copies	IID
FGF9	Copy number gain	4 copies	IID
MYC	Copy number gain	3 copies	IID
CCNE1	Copy number gain	5 copies	IIC

### Next generation sequencing of original tissue

The TruSight™ Oncology 500 (Illumina, USA) targeted, hybrid-capture based next-generation sequencing assay detected several mutations (variants of strong clinical significance and variants of potential clinical significance)—Level IB (*MYC, TP53*), IIC (*APC, BRCA1, CCND3*, *CCNE1, ERBB2, FGFR4, NOTCH1,* and *TP53*) and IID evidence (*AKT2, ASXL1, BRCA2, FGF9, PARP1* and *RET*), see Table 3 and Supplementary Fig. 6).

**Table 3:** The summary data and the sequencing statistics for whole exome sequencing analysis of Coala cancer cell line

A											
Chrom	Start	End	Ref	Alt	Func.Ref. Gene	GenRef. Gene	ExonicFunc.refGene	CLIN DIAGN	CLIN SIGN	DEPTH	VAF
1	56,940,965	56,940,965	G	A	Exonic	*C8B*	Stopgain	Complement_component_6_deficiency	Pathogenic	69	0.406
1	198,807,802	198,807,802	C	A	Downstream	*MIR181A1HG*	–	Acute_myeloid_leukemia_with_maturation	Pathogenic	56	0.232
1	198,826,991	198,826,991	C	T	ncRNA_intronic	*MIR181A1HG*	–	Acute_myeloid_leukemia_with_maturation	Pathogenic	22	0.227
1	198,898,549	198,898,549	G	T	ncRNA_intronic	*MIR181A1HG*	–	Acute_myeloid_leukemia_with_maturation	Pathogenic	33	1.0
1	198,898,955	198,898,955	G	A	ncRNA_intronic	*MIR181A1HG*	–	Acute_myeloid_leukemia_with_maturation	Pathogenic	30	1.0
1	198,900,385	198,900,385	T	C	ncRNA_intronic	*MIR181A1HG*	–	Acute_myeloid_leukemia_with_maturation	Pathogenic	43	1.0
10	6,485,181	6,485,181	G	A	Exonic	*PRKCQ*	Nonsynonymous SNV	Inflammatory_bowel_disease_1	Pathogenic	81	0.506
17	7,674,220	7,674,220	C	T	Exonic	*TP53*	Nonsynonymous SNV	Li-Fraumeni_syndrome_1|Hepatocellular_carcinoma|Neoplasm	Pathogenic	40	1.0
5	1,216,785	1,216,785	G	A	Intronic	*SLC6A19*	–	Hyperglycinurialiminoglycinuria_digenic	Pathogenic	135	0.185
5	112,839,515	112,839,519	AAAAG	–	Exonic	*APC*	Frameshift deletion	Gastric_polyposis|Duodenal_polyposis|Adenomatous_colonic	Pathogenic	66	0.394
5	150,848,436	150,848,436	C	T	Exonic	*IRGM*	Synonymous SNV	Inflammatory_bowel_disease_19	Pathogenic	72	0.431
5	177,093,242	177,093,242	G	A	Exonic	*FGFR4*	Nonsynonymous SNV	Cancer_progression_and_tumor_cell_motility	Pathogenic	57	0.439
6	43,520,078	43,520,078	G	A	Exonic	*POLR1C*	Nonsynonymous SNV	Leukodystrophy_hypomyelinating_ 11	Pathogenic	107	0.196
8	47,931,496	47,931,496	–	T	Intronic	*PRKDC*	–	Immunodeficiency_26_with_or_without_neurologic_abnormaliti	Pathogenic	60	0.2

B	
Total number of sequenced reads (in thousands)	32,222,600
Total number of uniquely mapped non-duplicate reads	28,259,220
Percentage of target bases with coverage > 30x	76%
Median coverage per targeted base	61
The percentage of targeted bases with coverage > 20	97.10%

### Pyrosequencing of Coala cell line

We analyzed 7CpGs for *MGMT*, 6CpGs for *CDKN2A*, 4CpGs for *PROM1* and 3CpGs for *ADAMTS16.* Out of four CRC biomarkers, three showed certain degree of hypomethylation. Percentage of methylation in each CpG island for every gene is shown in Supplementary Table 2. The percentage of methylation for *MGMT* was found to be 4% in CpG1, 2% in CpG2, 1% in CpG3, 3% in CpG4, 3% in CpG5, 3% in CpG6 and 9% in CpG7, whereas for *CDKN2A* it was 2% in CpG1, 3% in CpG2, 9% in CpG3, 5% in CpG4, 3% in CpG5 and 1% in CpG6. For *PROM1*, it was 1% in CpG1, 10% in CpG2, 1% in CpG3 and 4% in CpG4. The average percentage of methylation for unmethylated and methylated DNA was 1,85% (unmethylated) and 97.71% (methylated) for *MGMT*, 6% (unmethylated) and 72.3% (methylated) for *CDKN2A* and 1.5% (unmethylated) and 88.75% (methylated) for *PROM1*. Last CRC biomarker, *ADAMTS16*, showed a certain degree of hypermethylation in its selected region according to pyrosequencing analysis. The percentage of methylation was 98% in CpG1, 97% in CpG2 and 96% in CpG3. The unmethylated and methylated DNA showed average methylation to be 9% (unmethylated) and 92.3% (methylated) (Supplementary Table 2).

### Evaluation of metastatic potential

**Fig. 4:**
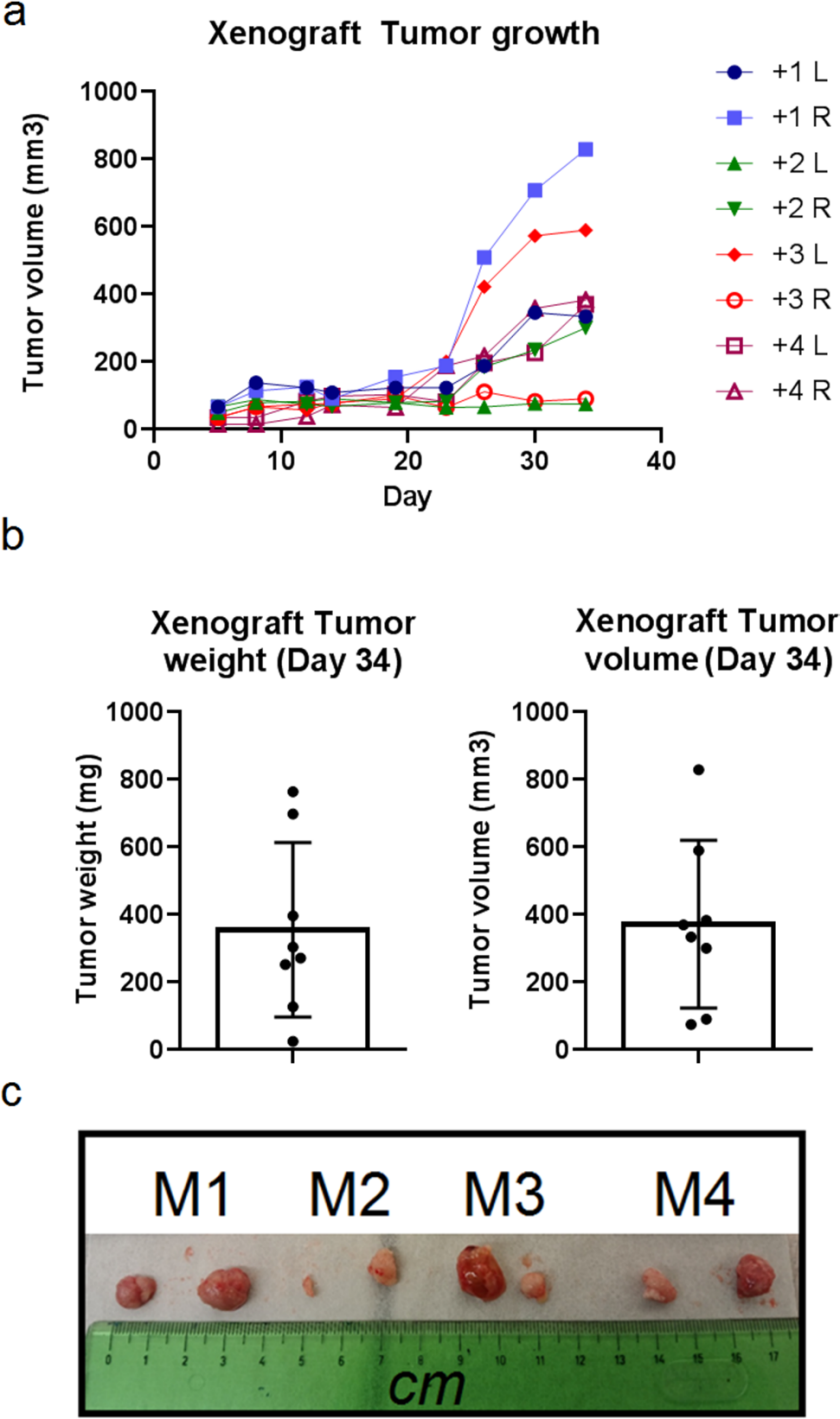
The analysis of Coala cancer cell line xenograft tumor growth (**a**), weight and volume (**b**) of tumors (**c**) in evaluation of metastatic potential of Coala cancer cell line. Cells from passage No.21 were used in experiment

**Fig. 5:**
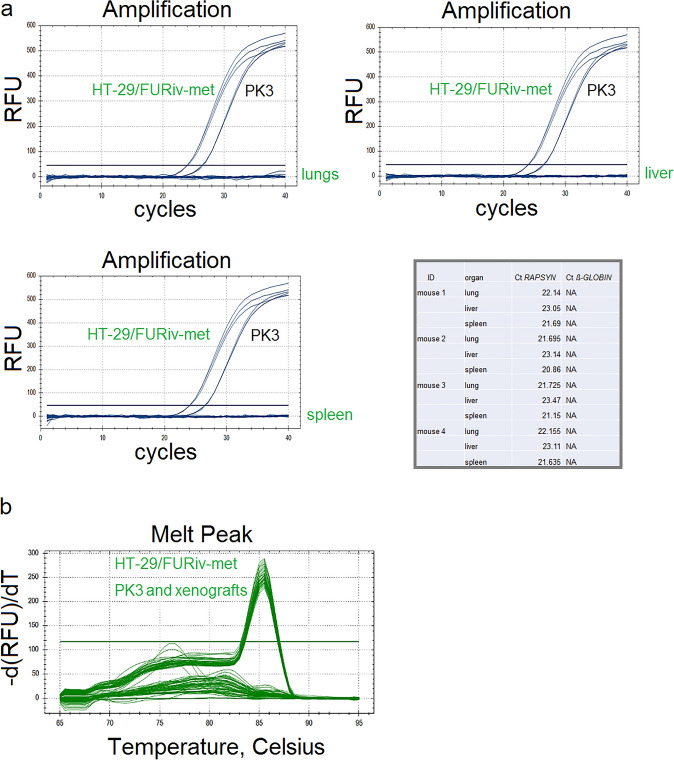
The qPCR analysis of human *β-GLOBIN* gene in samples from the lungs, livers and spleens of xenografted mice (**a**). Human *β-GLOBIN* was only detected in Coala cancer cell line-derived xenografts (**b**), confirming their human origin

During the growth of xenograft (34 days, Fig. 4a–c), mice showed no sign of cachexia or pain. During the autopsy, no visible metastases were detected. The lungs, livers, and spleens were subjected to detection of human *β-GLOBIN* gene by qPCR, which would point to the presence of micrometastases in the mouse tissue. As no human *β-GLOBIN* was detected in any lungs, livers, or spleens (Fig. 5a), we assumed that after 34 days, no micrometastases disseminated from Coala cancer cell line-induced subcutaneous xenografts. Human *β-GLOBIN* was only detected in Coala xenografts (XL means left xenograft, XR means right xenografts), confirming their human origin (Fig. 5b). The sequences of primers and probes used in the study are indicated in Supplementary Table 4.

## Discussion

The incidence of colorectal cancer has increased over the last decade [2]. Development of the new therapies for this cancer requires presence of adequate in vitro and in vivo models. In vitro models are typically represented by two-dimensional cell lines that are available for researchers via repository centers (like ATCC, Manassas, USA), or commercial companies. However, two-dimensional cell lines do not mimic the real human tumor three-dimensional architecture and therefore, do not represent the optimal model systems [13]. On the other hand, recent advances in 3D culture systems, tissue engineering and even 3D printing [14] allow researchers to use special form of culture conditions and grow cells in form of spheroid or organoids. Cells growing in such environment will assemble into structures that share some similarities with the real tumors [15]. Moreover, in future, these 3D culture systems will be probably preferred by pharmacological companies for new chemotherapy testing as tumor organoids have tendency to respond in the same way as real tumors [16]. Non-effective drugs are, therefore, pre-selected directly at the beginning of the new drug development process. The ability of cancer cells to form tumorspheres and grow in 3D is therefore another valuable characteristic of new in vitro model as some cancer cell lines do not have this ability and when placed in 3D culture, they only form atypical cell clumps.

Cancer cell lines retain many features of original tumors, but in the same time, they are known to acquire alternations (mutations) during the in vitro culture [17]. When genomic and expression profiles were compared by several research groups, it was found that some already existing cancer cell lines differ from the tumors of their origins and cannot be therefore considered as a really good models [17]. The next generation sequencing of our original tumor sample (liver metastasis) revealed several pathological mutations (Table 3, full list of the mutations is indicated in Supplementary Fig. 6), from them, three mutations (APC, TP53 and FGFR4) were also found to be conserved in Coala cancer cell line (Table 2 and Supplementary Table 1). Histology of the original tumor sample (liver metastasis) showed a weak signal for Cytokeratin 20 (Fig. 1f)*.* This is a well-known phenomenon, observed by many pathologists, that some tumor-specific antibodies show a weak signal (or no signal) at metastatic sites [18]. When fluorescent immunocytochemistry analysis was performed for Coala cells, it proved expression of *Cytokeratin 20* in this cell line (Fig. 2k, l)*.*

Multiplex flow cytometry analysis of all 376 human CD markers revealed expression of several surface markers that are associated with colorectal cancer. High expression (more than 90% of positive cells) was detected for several CD markers (e.g., CD 326 (*EpCAM*, one of the first cancer-associated antigens identified [19]), CD164 (promising new target for diagnosis and treatment of colon cancer [18]), CD147 (a potential 5-FU resistance biomarker for colorectal cancer patient [20]), CD98 (a potential prognostic biomarker for colorectal carcinoma [21]), CD29 (cell surface marker associated with cancer self-renewal [22]), CD26 (reported as a marker for colorectal cancer stem cells [23]) and CD9 to name a few) was detected. Interestingly, expression of CD9 (a member of tetraspanin superfamily, high expression of this tumor suppressor was connected to a favorable disease-free survival in colorectal cancer patients [24]) was comparable with expression of CD9 detected in SW480 colorectal cancer cells [25]. Interestingly, our newly developed Coala cancer cells express CD133 (10.99%). This CD marker is, according to several studies, one of the cancer stem cell markers [26, 27]. As many studies are now focused on research of rare cancer stem cell population, we believe that the new cancer cell line proposed by our group will become a useful research tool in the field.

Coala cell line was also tested for the sensitivity to 5-fluorouracil (Fig. 2n)*,* standard first-line treatment for colorectal cancer patients [28]. The sensitivity of Coala cell line to 5-fluorouracil after 48-h exposure was comparable to sensitivity of HT-29 colorectal carcinoma cells (HT-29 is an adherent epithelial cell line derived from patient with colorectal adenocarcinoma primary tumor) [29]. On the other hand, Coala cancer cell line shows a higher level of resistance when compared with two newly derived colorectal cancer cell lines, published recently (in 2024) by Cheng and co-authors [30]. This could be associated with the high expression of CD147 (a potential 5-FU resistance biomarker for colorectal cancer patient [20]).

Liver metastasis is the major contributor in colorectal cancer-related death. As the liver microenvironment take part in many steps of metastatic cascade from pre-metastatic niche formation, tumor cell colonization to metastatic tumor establishment, better understanding of mechanisms orchestrating the formation of a hepatic metastatic niche is necessary for the development of effective therapies. Until 2018, only a limited number of cell lines originated from human liver metastasis was reported by researchers [31–34]. Interestingly, no examples of 3D growth or tumorsphere formation were shown by authors of these studies. As already mentioned, the ability of cancer cell line or cancer cells to form organoids/tumorspheres is of interest in recent years as this feature is typically used to determine the percentage of cancer stem cells—a minor population of cancer cells with self-renewal and multi-lineage differentiation ability.

Recently published colorectal cancer cell lines (a pair of primary colorectal cancer-derived and corresponding synchronous liver-metastasis–derived organoid cell lines and patient-derived 3D cancer cell lines) were derived as organoid culture, capable of growing in form of 3D organoid structures [30]. These two cell lines were deposited into repository center (CCTCC, China Center for Type Culture Collection) for general use [30].

Interesting approach for utilizing the 3D culture to study metastatic colorectal cancer was used by D’Angelo et al. [35]. In this study, researchers showed an increased HT-29 cancer cell line proliferation and migration capability (together with some signs of epithelial–mesenchymal transition process) when cancer-derived scaffolds were used and compared with scaffold from liver tissue or healthy colon.

At ATCC (American Type Culture Collection), a collection of five patient-derived next generation cancer models—3D organoids (generated by the Human Cancer Models Initiative (HCMI) program) from primary tumor tissue of metastatic colon cancer is available to the researchers (Table 1). Five 2D cell lines derived from liver metastasis of colorectal cancer patient were deposited at the Leibniz Institute DSMZ (Deutsche Sammlung von Mikroorganismen und Zellkulturen) (Table 1). In 2025, two new 3D organoid cell lines were derived and deposited into repository center (CCTCC, China Center for Type Culture Collection) for general use [30]. Therefore, the lack of established and well-characterized 2D/3D cancer cell lines, derived from liver metastatic tissue of colorectal patients still remains.

In conclusion, Coala cancer cell line represents a new cancer cell line model and will be available to the researchers and research groups via local and international repository centers.

## Supplementary Information

Below is the link to the electronic supplementary material.

Supplementary Fig.1 The chromosomes derived from cultured Coala cells, analyzed by standard cytogenetic G-banding method (TIF 102 KB)

Supplementary Fig.2 The list of antibodies, used in the study (TIF 53 KB)

Supplementary Fig.3 Multiplex flow cytometry analysis of all 376 human surface CD markers (both positive and negative CD markers are show) (TIFF 2469 KB)

Supplementary Fig.4 Multiplex flow cytometry analysis of positively-stained CD markers of newly derived Coala cells. Image (heat map) shows the percentage of positive cells detected for each CD marker. Negative CD markers are not included (TIF 1329 KB)

Supplementary Fig.5 Selected loci from STR fingerprinting analysis confirming the same origin of tumor sample and isolated Coala cell line. Cells from passage No.21 were used (TIF 421 KB)

Supplementary Fig.6 The list of mutations found in the original metastasis with The TruSight Oncology 500 (Illumina, USA) targeted; hybrid-capture based next-generation sequencing assay (TIF 315 KB)

Supplementary Table 1 (XLSX 48414 KB)

Supplementary Table 2 (TIF 968 KB)

Supplementary Table 3 (TIF 2902 KB)

Supplementary Table 4 (TIF 54 KB)

## Data Availability

The data generated during the current study and used to support the findings are available from the corresponding authors upon reasonable request.

## Declarations

### The use of animals in the project

Animals were maintained in accordance with the standards of the Jessenius Faculty of Medicine in Martin, Comenius University in Bratislava, Slovak Republic. Animal experiments were approved by Ethical Review Committee of Jessenius Faculty of Medicine in Martin, Comenius University in Bratislava, The State Veterinary and Food Administration of the Slovak Republic (Approval No. 4987-3/2022-220 and No: Ro-290-3/2020-220).

## References

[1] Jones P, Cade JE, Evans CEL, Hancock N, Greenwood DC. Does adherence to the World Cancer Research Fund/American Institute of Cancer Research cancer prevention guidelines reduce risk of colorectal cancer in the UK women’s cohort study? Br J Nutr. 2018;119(3):340–8. 10.1017/S0007114517003622. (**Epub 2018 Jan 21. PMID: 29352814**). 29352814 10.1017/S0007114517003622

[2] Morgan E, Arnold M, Gini A, et al. Global burden of colorectal cancer in 2020 and 2040: incidence and mortality estimates from GLOBOCAN. Gut. 2023;72(2):338–44. 10.1136/gutjnl-2022-327736. (**Epub 2022 Sep 8. PMID: 36604116**). 36604116 10.1136/gutjnl-2022-327736

[3] Siegel RL, Kratzer TB, Giaquinto AN, Sung H, Jemal A. Cancer statistics, 2025. CA Cancer J Clin. 2025;75(1):10–45. 10.3322/caac.21871. (**Epub 2025 Jan 16. PMID: 39817679; PMCID: PMC11745215**). 39817679 10.3322/caac.21871 PMC11745215

[4] Hess KR, Varadhachary GR, Taylor SH, Wei W, Raber MN, Lenzi R, Abbruzzese JL. Metastatic patterns in adenocarcinoma. Cancer. 2006;106(7):1624–33. 10.1002/cncr.21778. (**PMID: 16518827**). 16518827 10.1002/cncr.21778

[5] Chaffer CL, San Juan BP, Lim E, Weinberg RA. EMT, cell plasticity and metastasis. Cancer Metastasis Rev. 2016;35(4):645–54. 27878502 10.1007/s10555-016-9648-7

[6] Lau HCH, Kranenburg O, Xiao H, Yu J. Organoid models of gastrointestinal cancers in basic and translational research. Nat Rev Gastroenterol Hepatol. 2020;17(4):203–22. 10.1038/s41575-019-0255-2. (**Epub 2020 Feb 25. PMID: 32099092**). 32099092 10.1038/s41575-019-0255-2

[7] Mo S, Tang P, Luo W, Zhang L, Li Y, Hu X, Ma X, Chen Y, Bao Y, He X, Fu G, Xu X, Rao X, Li X, Guan R, Chen S, Deng Y, Lv T, Mu P, Zheng Q, Wang S, Liu F, Li Y, Sheng W, Huang D, Hu C, Gao J, Zhang Z, Cai S, Clevers H, Peng J, Hua G. Patient-derived organoids from colorectal cancer with paired liver metastasis reveal tumor heterogeneity and predict response to chemotherapy. Adv Sci (Weinh). 2022;9(31): e2204097. 10.1002/advs.202204097. (**Epub 2022 Sep 4. PMID: 36058001; PMCID: PMC9631073**). 36058001 10.1002/advs.202204097 PMC9631073

[8] Bruun J, Kryeziu K, Eide PW, et al. Patient-derived organoids from multiple colorectal cancer liver metastases reveal moderate intra-patient pharmacotranscriptomic heterogeneity. Clin Cancer Res. 2020;26(15):4107–19. 10.1158/1078-0432.CCR-19-3637. 32299813 10.1158/1078-0432.CCR-19-3637

[9] Pham PT, Pekarcikova J, Edelstein R, Majdan M. Joinpoint analysis of colorectal cancer trend in the Slovakia. Bratisl Lek Listy. 2023;124(11):833–41. 10.4149/BLL_2023_128. (**PMID: 37874806**). 37874806 10.4149/BLL_2023_128

[10] Hashiguchi Y, Muro K, Saito Y, et al., Japanese Society for Cancer of the Colon and Rectum. Japanese society for cancer of the colon and rectum (JSCCR) guidelines 2019 for the treatment of colorectal cancer. Int J Clin Oncol. 2020;25(1):1–42. 10.1007/s10147-019-01485-z. (**Epub 2019 Jun 15. PMID: 31203527; PMCID: PMC6946738**). 10.1007/s10147-019-01485-z PMC6946738 31203527

[11] Strnadel J, Woo SM, Choi S, Wang H, Grendar M, Fujimura K. 3D culture protocol for testing gene knockdown efficiency and cell line derivation. Bio-Protoc. 2018;8(11): e2874. 10.21769/BioProtoc.2874. 34285988 10.21769/BioProtoc.2874 PMC8275222

[12] Zahumenska R, Kalman M, Marcinek J, et al. Establishment of PANDA—a new human pancreatic ductal adenocarcinoma cell line with 3D cell culture technology. Neoplasma. 2022;69(1):165–73. 10.4149/neo_2021_210924N1360. (**Epub 2021 Nov 25. PMID: 34818028**). 34818028 10.4149/neo_2021_210924N1360

[13] Sun Y, Ma H. Application of three-dimensional cell culture technology in screening anticancer drugs. Biotechnol Lett. 2023;45(9):1073–92. 10.1007/s10529-023-03410-x. (**PMID: 37421554**). 37421554 10.1007/s10529-023-03410-x

[14] Safhi AY. Three-dimensional (3D) printing in cancer therapy and diagnostics: current status and future perspectives. Pharmaceuticals (Basel). 2022;15(6): 678. 10.3390/ph15060678. (**PMID: 35745597; PMCID: PMC9229198**). 35745597 10.3390/ph15060678 PMC9229198

[15] Barbosa MAG, Xavier CPR, Pereira RF, Petrikaitė V, Vasconcelos MH. 3D cell culture models as recapitulators of the tumor microenvironment for the screening of anti-cancer drugs. Cancers (Basel). 2021;14(1): 190. 10.3390/cancers14010190. (**PMID: 35008353; PMCID: PMC8749977**). 35008353 10.3390/cancers14010190 PMC8749977

[16] Sztankovics D, Moldvai D, Petővári G, et al. 3D bioprinting and the revolution in experimental cancer model systems-a review of developing new models and experiences with *in vitro* 3D bioprinted breast cancer tissue-mimetic structures. Pathol Oncol Res. 2023;29: 1610996. 10.3389/pore.2023.1610996. (**PMID: 36843955; PMCID: PMC9946983**). 36843955 10.3389/pore.2023.1610996 PMC9946983

[17] Sinha R, Luna A, Schultz N, Sander C. A pan-cancer survey of cell line tumor similarity by feature-weighted molecular profiles. Cell Rep Methods. 2021;1(2): 100039. 10.1016/j.crmeth.2021.100039. (**PMID: 35475239; PMCID: PMC9017219**). 35475239 10.1016/j.crmeth.2021.100039 PMC9017219

[18] Selves J, Long-Mira E, Mathieu MC, Rochaix P, Ilié M. Immunohistochemistry for diagnosis of metastatic carcinomas of unknown primary site. Cancers (Basel). 2018;10(4): 108. 10.3390/cancers10040108. (**PMID: 29621151; PMCID: PMC5923363**). 29621151 10.3390/cancers10040108 PMC5923363

[19] Baeuerle PA, Gires O. EpCAM (CD326) finding its role in cancer. Br J Cancer. 2007;96(3):417–23. 10.1038/sj.bjc.6603494. (**PMID: 17211480; PMCID: PMC2360029**). 17211480 10.1038/sj.bjc.6603494 PMC2360029

[20] Tang J, Zhang L, She X, Zhou G, Yu F, Xiang J, Li G. Inhibiting CD164 expression in colon cancer cell line HCT116 leads to reduced cancer cell proliferation, mobility, and metastasis in vitro and in vivo. Cancer Investig. 2012;30(5):380–9. 10.3109/07357907.2012.666692. (**Epub 2012 Mar 12. PMID: 22409183**). 22409183 10.3109/07357907.2012.666692

[21] Dong S, Li S, Wang X, et al. CD147 mediates 5-fluorouracil resistance in colorectal cancer by reprogramming glycolipid metabolism. Front Oncol. 2022;12: 813852. 10.3389/fonc.2022.813852. (**PMID: 35898887; PMCID: PMC9309564**). 35898887 10.3389/fonc.2022.813852 PMC9309564

[22] Cherciu I, Bărbălan A, Pirici D, Mărgăritescu C, Săftoiu A. Stem cells, colorectal cancer and cancer stem cell markers correlations. Curr Health Sci J. 2014;40(3):153–61. 10.12865/CHSJ.40.03.01. (**Epub 2014 Aug 4. PMID: 25729599; PMCID: PMC4340434**). 25729599 10.12865/CHSJ.40.03.01 PMC4340434

[23] Ng L, Wong SK, Huang Z, et al. CD26 induces colorectal cancer angiogenesis and metastasis through CAV1/MMP1 signaling. Int J Mol Sci. 2022;23(3): 1181. 10.3390/ijms23031181. (**PMID: 35163100; PMCID: PMC8835326**). 35163100 10.3390/ijms23031181 PMC8835326

[24] Kim KJ, Kwon HJ, Kim MC, Bae YK. CD9 expression in colorectal carcinomas and its prognostic significance. J Pathol Transl Med. 2016;50(6):459–68. 10.4132/jptm.2016.10.02. (**Epub 2016 Oct 25. PMID: 27780340; PMCID: PMC5122733**). 27780340 10.4132/jptm.2016.10.02 PMC5122733

[25] Orchard-Webb DJ, Lee TC, Cook GP, Blair GE. CUB domain containing protein 1 (CDCP1) modulates adhesion and motility in colon cancer cells. BMC Cancer. 2014;14: 754. 10.1186/1471-2407-14-754. (**PMID: 25301083; PMCID: PMC4200232**). 25301083 10.1186/1471-2407-14-754 PMC4200232

[26] Akbari M, Shomali N, Faraji A, Shanehbandi D, Asadi M, Mokhtarzadeh A, Shabani A, Baradaran B. CD133: an emerging prognostic factor and therapeutic target in colorectal cancer. Cell Biol Int. 2020;44(2):368–80. 10.1002/cbin.11243. (**Epub 2019 Oct 18. PMID: 31579983**). 31579983 10.1002/cbin.11243

[27] Guan S, Yang R, Wu S, Xu K, Yang C. The CD133^+^CXCR4^+^ colorectal tumor cells promote colorectal cancer progression by PI3K/AKT signaling. J Interf Cytokine Res. 2022;42(5):195–202. 10.1089/jir.2021.0207. (**Epub 2022 Apr 4. PMID: 35377243**). 10.1089/jir.2021.0207 35377243

[28] Huang X, Ke K, Jin W, et al. Identification of genes related to 5-fluorouracil based chemotherapy for colorectal cancer. Front Immunol. 2022;13: 887048. 10.3389/fimmu.2022.887048. (**PMID: 35784334; PMCID: PMC9247273**). 35784334 10.3389/fimmu.2022.887048 PMC9247273

[29] Sasaki K, Tsuno NH, Sunami E, et al. Chloroquine potentiates the anti-cancer effect of 5-fluorouracil on colon cancer cells. BMC Cancer. 2010;10(1): 370. 10.1186/1471-2407-10-370. (**PMID: 20630104; PMCID: PMC2914703**). 20630104 10.1186/1471-2407-10-370 PMC2914703

[30] Cheng F, Li P, Xu S, Zhang C, Liang H, Ding Z. A pair of primary colorectal cancer-derived and corresponding synchronous liver metastasis-derived organoid cell lines. Aging (Albany NY). 2024;16(5):4396–422. 10.18632/aging.205595. (**Epub 2024 Feb 24. PMID: 38407980; PMCID: PMC10968669**). 38407980 10.18632/aging.205595 PMC10968669

[31] Yamachika T, Nakanishi H, Yasui K, Ikehara Y, Niwa T, Wanibuchi H, Tatematsu M, Fukushima S. Establishment and characterization of a human colonic mucinous carcinoma cell line with predominant goblet-cell differentiation from liver metastasis. Pathol Int. 2005;55(9):550–7. 10.1111/j.1440-1827.2005.01868.x. (**PMID: 16143030**). 16143030 10.1111/j.1440-1827.2005.01868.x

[32] Chai Y, Wang H, Zhou F. Establishment and characterization of a cell line HCS1220 from human liver metastasis of colon cancer. Cancer Cell Int. 2018;18(1): 137. 10.1186/s12935-018-0630-z. (**PMID: 30214379; PMCID: PMC6131799**). 30214379 10.1186/s12935-018-0630-z PMC6131799

[33] Rowehl RA, Burke S, Bialkowska AB, Pettet DW 3rd, Rowehl L, Li E, Antoniou E, Zhang Y, Bergamaschi R, Shroyer KR, Ojima I, Botchkina GI. Establishment of highly tumorigenic human colorectal cancer cell line (CR4) with properties of putative cancer stem cells. PLoS ONE. 2014;9(6): e99091. 10.1371/journal.pone.0099091. (**PMID: 24921652; PMCID: PMC4055451**). 24921652 10.1371/journal.pone.0099091 PMC4055451

[34] Boot A, van Eendenburg J, Crobach S, Ruano D, Speetjens F, Calame J, Oosting J, Morreau H, van Wezel T. Characterization of novel low passage primary and metastatic colorectal cancer cell lines. Oncotarget. 2016;7(12):14499–509. 10.18632/oncotarget.7391. (**PMID: 26894854; PMCID: PMC4924731**). 26894854 10.18632/oncotarget.7391 PMC4924731

[35] D’Angelo E, Natarajan D, Sensi F, Ajayi O, Fassan M, Mammano E, Pilati P, Pavan P, Bresolin S, Preziosi M, Miquel R, Zen Y, Chokshi S, Menon K, Heaton N, Spolverato G, Piccoli M, Williams R, Urbani L, Agostini M. Patient-derived scaffolds of colorectal cancer metastases as an organotypic 3D model of the liver metastatic microenvironment. Cancers (Basel). 2020;12(2):364. 10.3390/cancers12020364. (**PMID: 32033473; PMCID: PMC7072130**). 32033473 10.3390/cancers12020364 PMC7072130

