# Supplementary figures and images for: Establishment of Coala: a novel 3D and 2D cancer cell line derived from colorectal cancer liver metastasis
Source: Hum Cell. 2025 Aug 4;38(5):137. doi: 10.1007/s13577-025-01256-1 (PMC12321644; doi:10.1007/s13577-025-01256-1)

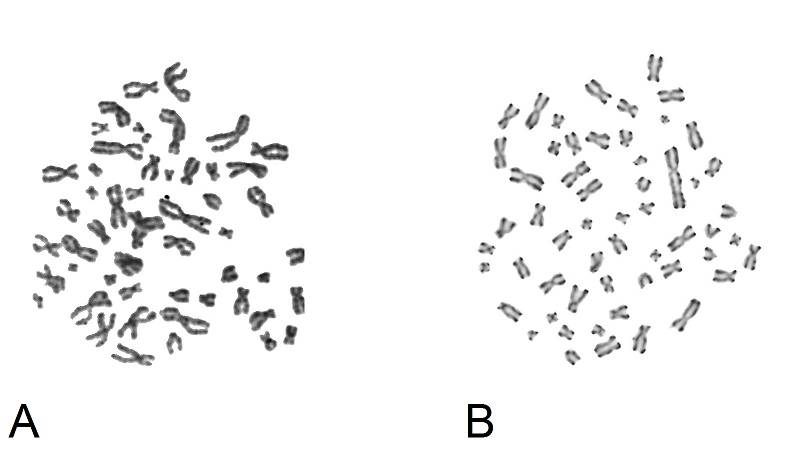

Supplement: Supplementary file 1 — Supplementary Fig.1 The chromosomes derived from cultured Coala cells, analyzed by standard cytogenetic G-banding method (TIF 102 KB) [file 13577_2025_1256_MOESM1_ESM.tif]

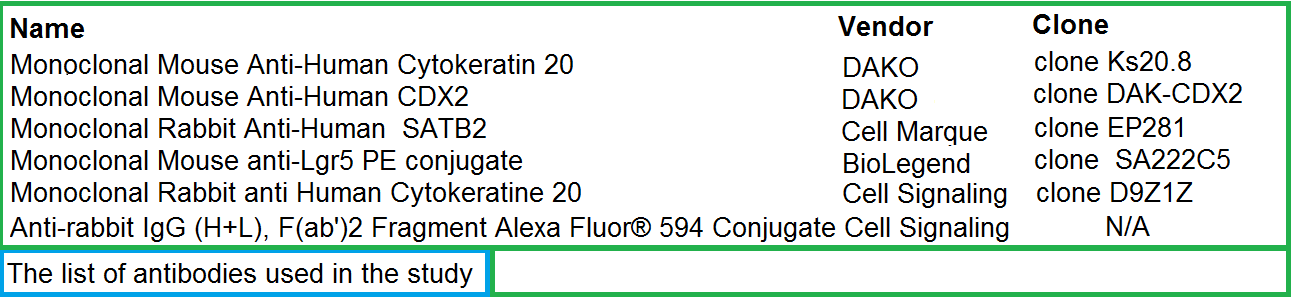

Supplement: Supplementary file 2 — Supplementary Fig.2 The list of antibodies, used in the study (TIF 53 KB) [file 13577_2025_1256_MOESM2_ESM.tif]

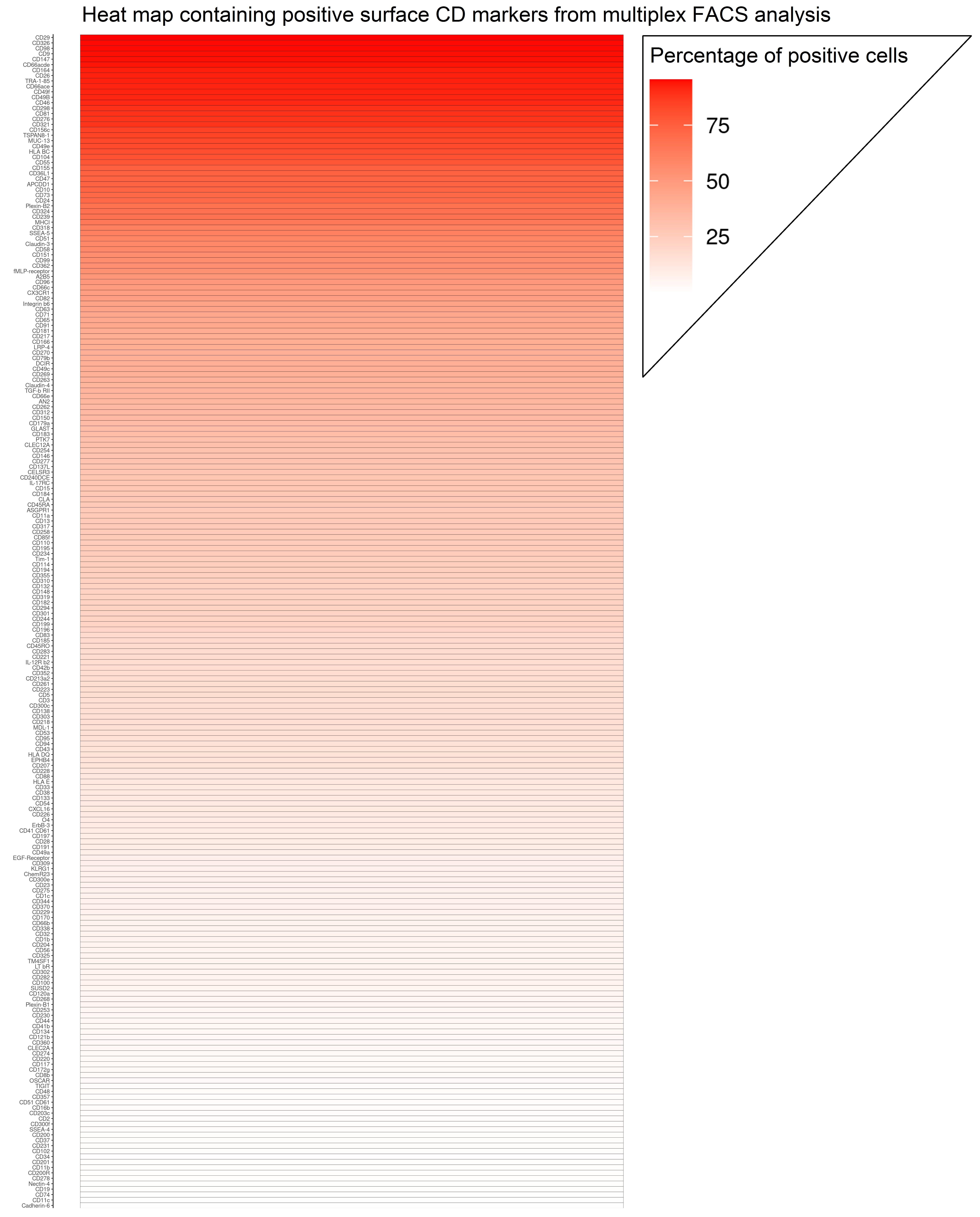

Supplement: Supplementary file 4 — Supplementary Fig.4 Multiplex flow cytometry analysis of positively-stained CD markers of newly derived Coala cells. Image (heat map) shows the percentage of positive cells detected for each CD marker. Negative CD markers are not included (TIF 1329 KB) [file 13577_2025_1256_MOESM4_ESM.tif]

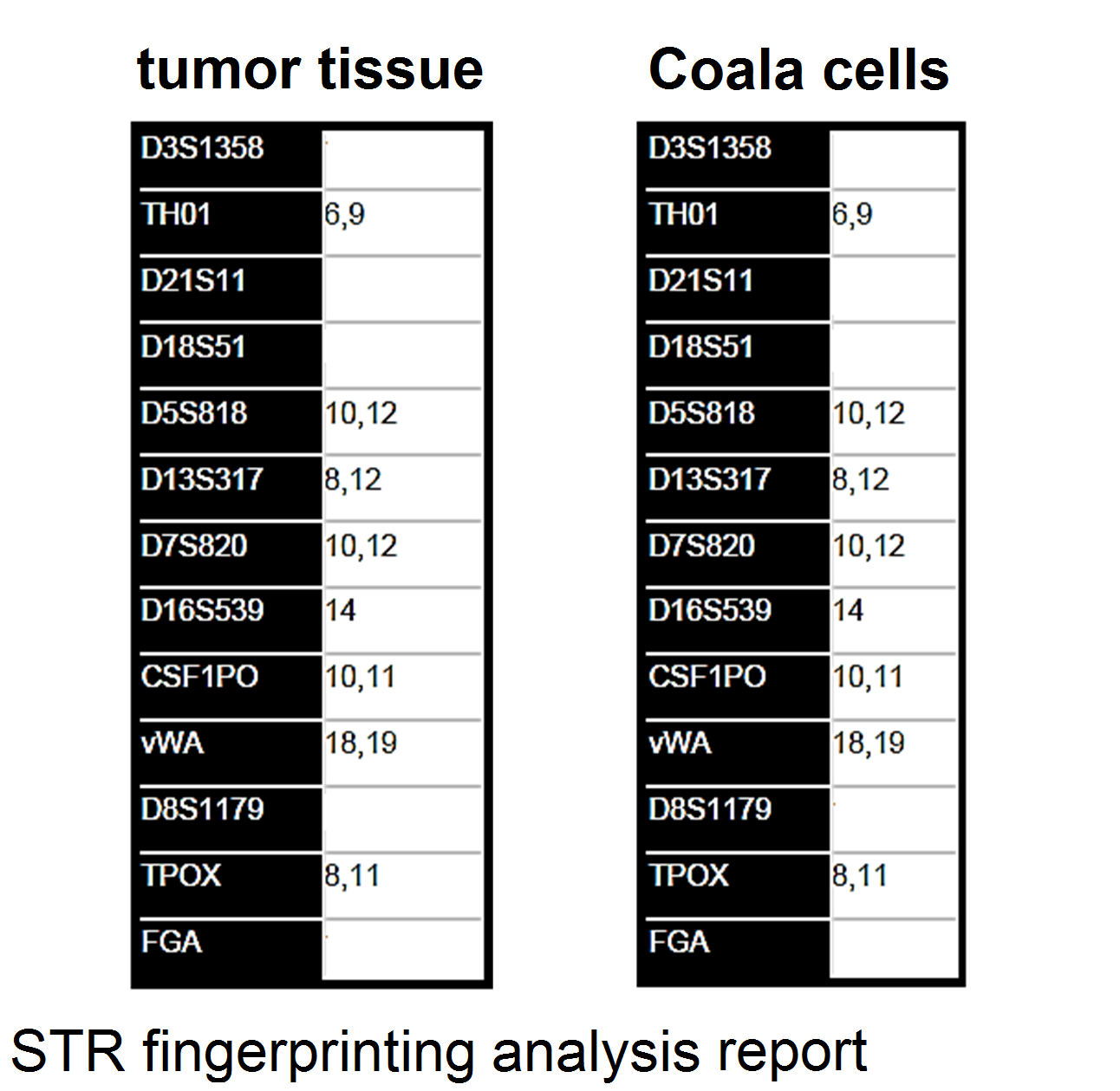

Supplement: Supplementary file 5 — Supplementary Fig.5 Selected loci from STR fingerprinting analysis confirming the same origin of tumor sample and isolated Coala cell line. Cells from passage No.21 were used (TIF 421 KB) [file 13577_2025_1256_MOESM5_ESM.tif]

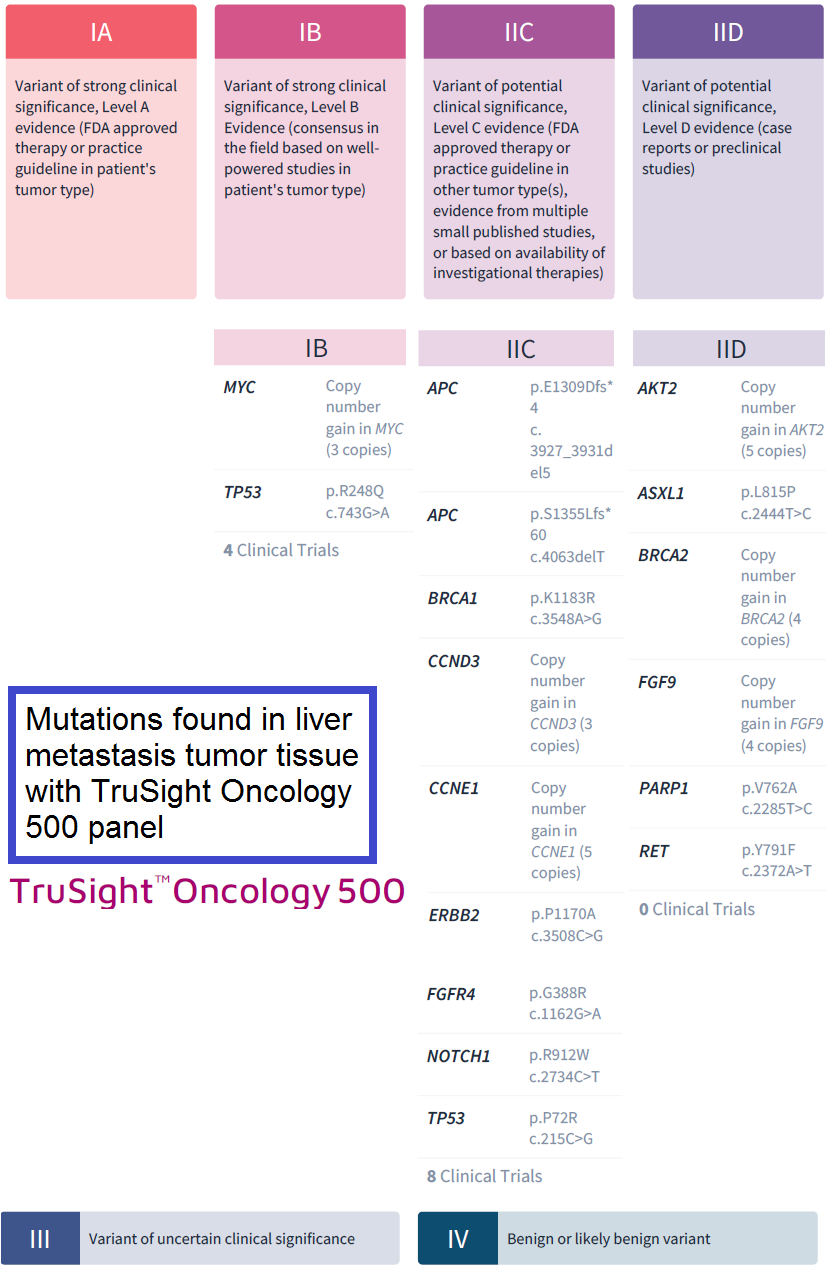

Supplement: Supplementary file 6 — Supplementary Fig.6 The list of mutations found in the original metastasis with The TruSight Oncology 500 (Illumina, USA) targeted; hybrid-capture based next-generation sequencing assay (TIF 315 KB) [file 13577_2025_1256_MOESM6_ESM.tif]

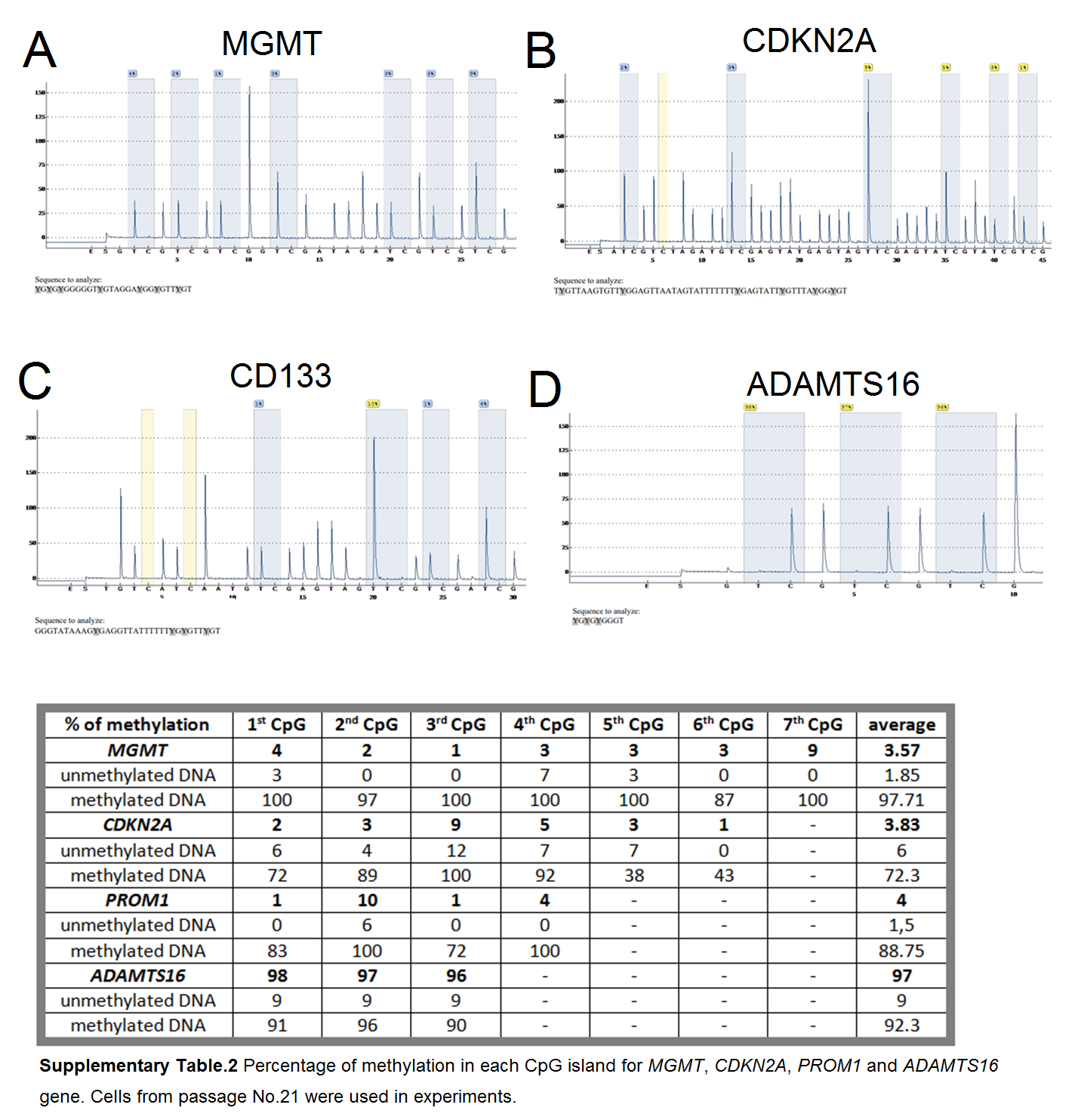

Supplement: Supplementary file 8 — Supplementary Table 2 (TIF 968 KB) [file 13577_2025_1256_MOESM8_ESM.tif]

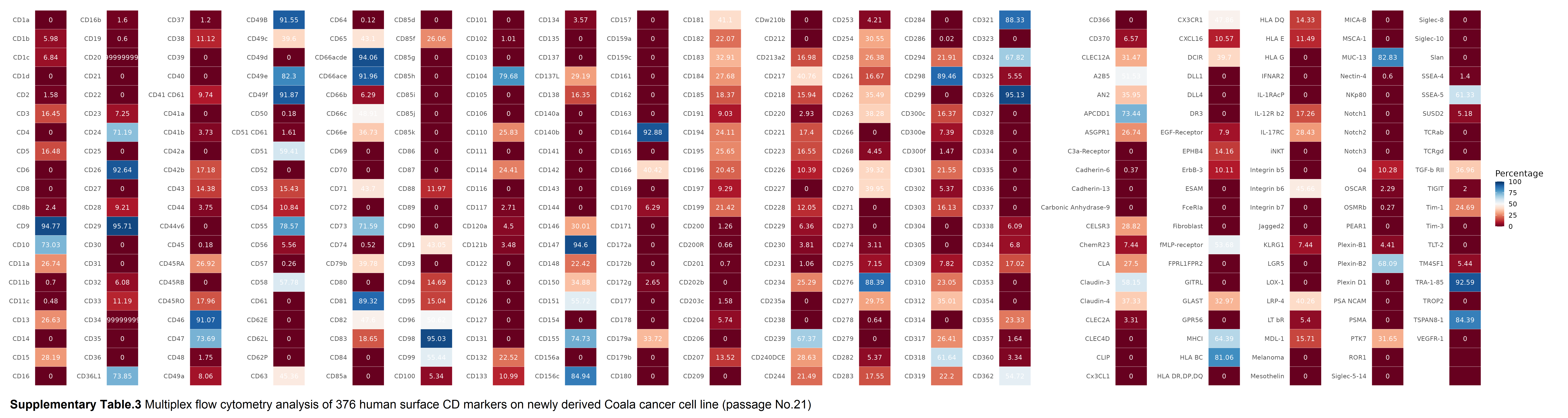

Supplement: Supplementary file 9 — Supplementary Table 3 (TIF 2902 KB) [file 13577_2025_1256_MOESM9_ESM.tiff]

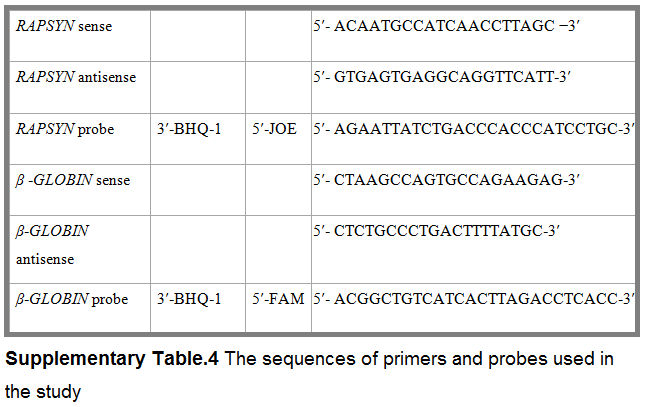

Supplement: Supplementary file 10 — Supplementary Table 4 (TIF 54 KB) [file 13577_2025_1256_MOESM10_ESM.tif]
